# MRSA Transmission on a Neonatal Intensive Care Unit: Epidemiological and Genome-Based Phylogenetic Analyses

**DOI:** 10.1371/journal.pone.0054898

**Published:** 2013-01-31

**Authors:** Ulrich Nübel, Matthias Nachtnebel, Gerhard Falkenhorst, Justus Benzler, Jochen Hecht, Michael Kube, Felix Bröcker, Karin Moelling, Christoph Bührer, Petra Gastmeier, Brar Piening, Michael Behnke, Manuel Dehnert, Franziska Layer, Wolfgang Witte, Tim Eckmanns

**Affiliations:** 1 Department of Infectious Diseases, Unit of Nosocomial Infections, Robert Koch Institute, Wernigerode, Germany; 2 Department for Infectious Disease Epidemiology, Robert Koch Institute, Berlin, Germany; 3 Post Graduate Training in Applied Epidemiology, Robert Koch Institute, Berlin, Germany; 4 European Programme for Intervention Epidemiology Training (EPIET), European Centre for Disease Prevention and Control (ECDC), Stockholm, Sweden; 5 Max Planck Institute for Molecular Genetics, Berlin, Germany; 6 Berlin-Brandenburg Center for Regenerative Therapies, Charité University Medical Center, Berlin, Germany; 7 Institute of Medical Virology, University of Zürich, Zürich, Switzerland; 8 Department of Neonatology, Charité University Medical Center, Berlin, Germany; 9 Institute of Hygiene and Environmental Medicine, Charité University Medical Center, Berlin, Germany; Rockefeller University, United States of America

## Abstract

**Background:**

Methicillin-resistant *Staphylococcus aureus* (MRSA) may cause prolonged outbreaks of infections in neonatal intensive care units (NICUs). While the specific factors favouring MRSA spread on neonatal wards are not well understood, colonized infants, their relatives, or health-care workers may all be sources for MRSA transmission. Whole-genome sequencing may provide a new tool for elucidating transmission pathways of MRSA at a local scale.

**Methods and Findings:**

We applied whole-genome sequencing to trace MRSA spread in a NICU and performed a case-control study to identify risk factors for MRSA transmission. MRSA genomes had accumulated sequence variation sufficiently fast to reflect epidemiological linkage among individual patients, between infants and their mothers, and between infants and staff members, such that the relevance of individual nurses’ nasal MRSA colonization for prolonged transmission could be evaluated. In addition to confirming previously reported risk factors, we identified an increased risk of transmission from infants with as yet unknown MRSA colonisation, in contrast to known MRSA-positive infants.

**Conclusions:**

The integration of epidemiological (temporal, spatial) and genomic data enabled the phylogenetic testing of several hypotheses on specific MRSA transmission routes within a neonatal intensive-care unit. The pronounced risk of transmission emanating from undetected MRSA carriers suggested that increasing the frequency or speed of microbiological diagnostics could help to reduce transmission of MRSA.

## Introduction

Methicillin-resistant *Staphylococcus aureus* (MRSA) may cause prolonged outbreaks of infections in neonatal intensive care units (NICUs), which may require aggressive, multi-faceted infection control measures [1]–[4]. Infants who weigh <1,500 g at birth (very low birth weight [VLBW] infants) are most vulnerable to serious MRSA infections [5]. Clinical cultures have been reported to underestimate MRSA colonization in NICUs, whereas active surveillance cultures could detect MRSA-affected infants earlier and thus limit nosocomial spread [6]. MRSA screening, however, is associated with increased costs and may lead to problems related to false-positive results and unintended consequences [7]. While the specific factors favouring MRSA transmission on neonatal wards are not well understood, health-care workers, other patients cared for by the same medical personnel, and family members including the patients’ mothers or siblings, may be sources of MRSA colonization [8].

Genotyping of MRSA isolates has assisted investigations of MRSA spread within and among hospitals [9], [10]. A variety of molecular methods has been applied to differentiate and track strains of MRSA, but all these approaches provide limited discriminatory power at a local scale, where single variants commonly predominate the pathogen population [9], [11]–[13]. In contrast, epidemiological linkage between individual patients can be tested phylogenetically for pathogens that accumulate nucleic acid variation over sufficiently short timescales [14]–[17]. While this concept and associated analysis tools were applied to rapidly evolving RNA viruses in the past, it was established only recently that MRSA may constitute such “measurably evolving populations”, suggesting that sequencing MRSA genomes may provide a new tool to elucidate transmission chains and pathogen reservoirs [18], [19]. Two recent papers reported that MRSA whole-genome sequencing was able to distinguish outbreak strains from unrelated strains within the same hospital. Importantly, such sequence data could be generated and analysed quickly enough to impact on patient care [20], [21].

Here, we demonstrate the utility of MRSA genome sequencing to infer the transmission history of MRSA in a NICU. MRSA genomes proved to be highly informative for supporting a case-control study that was performed to identify risk factors for transmission of MRSA.

## Methods

### Setting

The study was conducted in the neonatology unit of a tertiary care hospital in Berlin, comprising three wards with a total capacity of 56 beds. The retrospective case-control study spanned the period February 8th to August 31st, 2010. Screening of all admitted infants by nasopharyngeal and perianal swabbing for MRSA culture was performed once a week from February 8th, 2010, and twice weekly from July 21st, 2010 until the end of our study. In addition, 166 staff members were screened by nasopharyngeal swabbing in February and August 2010.

Because our investigation was commissioned by the local health department (Gesundheitsamt Berlin Mitte) in accordance with article 25 paragraph 1 of the German Infection Protection Act of 2001, and in agreement with the responsible ethical review board (Ethics Commission Charité - Universitätsmedizin Berlin), a formal ethical review process and approval was not required to meet compliance with the Declaration of Helsinki, and consent from the next of kin, caretakers, or guardians of the neonates was not needed.

### Case-control Study

To identify risk factors for MRSA transmission, we conducted a retrospective matched case-control study. We defined a case as a patient in the NICU in whom colonization or infection with MRSA *spa* type *t032* (multilocus sequence type ST22) was detected between February 8th and August 31st, 2010. The presumptive exposure period for MRSA transmission was from birth or one day before the last negative swab to one day before the first positive swab. Controls were MRSA-negative NICU patients, matched for birth weight (+/−100 g). If more than two eligible controls were identified from the inpatient registry, two were randomly selected. Cases and controls were not matched by date of admission to avoid over-matching for possible time-dependent factors affecting an entire ward, e.g. presence of a colonized staff member. In addition to basic data like mode of delivery, length of hospitalisation etc. (Table 1), we compared a wide range of exposures in the presumed exposure period of each case and in the corresponding days of life of the controls, including type of nutrition, antibiotics, other oral drugs, blood transfusions, gastric tube, i.v. lines, urine catheter, type of ventilation/endotracheal intubation, suction of airways, surgical operations, other invasive procedures, episodes of bradycardia and physical stimulations, incubator/warming bed, ultrasound examinations, X-rays, ECG, hearing tests, other specialist examinations, physiotherapy, skin-to-skin (‘kangaroo’) care, names of nursing staff and physicians caring for patient, ward and room for each day of exposure period, body weight on day of MRSA detection. Nursing staff and physicians caring for each patient were identified based on duty rosters for each day and ward. The infants-to-nurse ratio was calculated as the average number of infants admitted to the same ward during the exposure period divided by the average number of nurses on duty.

**Table 1:** Characteristics of the cases and controls matched for weight at birth and age during exposure time.

	Cases	Controls	Level of significance (p-value)#
Weight at birth (median and range)	1165 g (606–3800 g)	1256 g‡ (625–3740 g)	0.91
MRSA infection†	22% (5/23)	n/a	
Duration until MRSA positive (median and range)	8 days* (2–91 days)	n/a	
Male gender	52% (12/23)	41% (15/37)	0.38
Birth by caesarean section	83% (19/23)	81% (29/36)	0.84
Multiples	52% (12/23)	35% (13/37)	0.15
Gestational age (median and range)	29 weeks (23–42)	32 weeks‡ (24–41)	0.43
Born on-site	91% (21/23)	97% (32/33)	0.35
Length of stay (median and range)	47 days (6–103)	38 days‡ (7–116)	0.61

* from birth or last negative swab to first positive.

# Kruskal Wallis, Chi2.

† as opposed to colonisation.

‡ In pairs with two controls, the average value of the controls was used for the calculation.

**Figure 1:**
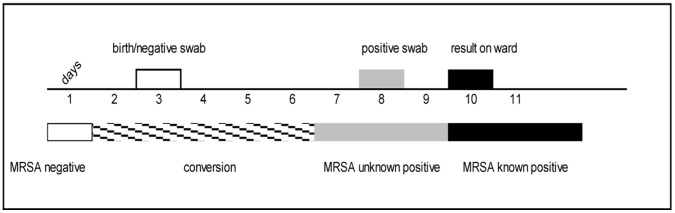
Definition of MRSA-related patient status. Initially, birth or a negative swab result in the status “MRSA-negative”. A few days later another swab is taken, which turns out MRSA-positive. MRSA is presumed to have been acquired latest one day before the positive swab was taken, because it takes time for the bacteria to multiply and spread from the location of transmission to the location being swabbed. Therefore, the infant’s status is “unknown MRSA-positive” from one day before the positive swab until the positive result is received on the ward. Thereafter, the infant’s status is “known MRSA-positive”.

We defined an infant who was considered MRSA-negative on the basis of available test results, but who in fact was already MRSA-positive, as “unknown MRSA-positive”. At the time when a positive swab result was received on the ward (on average two days after swabbing), an infant turned to status “known MRSA-positive” (Figure 1).

Descriptive statistics comprised the calculation of median and ranges for continuous variables, and absolute numbers and proportions for categorical variables. Comparative analyses were performed based on Kruskal Wallis test and univariable exact logistic regression for matched analyses. All reported p-values are two-sided and p<0.05 was considered significant. Statistical analyses were performed with Stata 11.0 (StataCorp LP, TX, USA).

### MRSA Genome Sequencing and Analyses

Staphylococcal colonies on agar plates were randomly selected for analysis. Following DNA extraction, multiplexed sequencing of genomic DNA from *S. aureus* isolates was performed on an Illumina GAIIx sequencer, providing 100-fold average coverage. Single-nucleotide polymorphisms (SNPs) were identified by mapping paired-end sequencing reads against the genome sequence from a related ST22 isolate [21] and subsequently verified through dedicated PCRs and capillary sequencing. An alignment of SNPs in the non-repetitive core genome was used to reconstruct the isolates’ phylogeny by applying PhyML 3.0.1 and to calculate evolutionary rates and divergence times with the BEAST software (http://beast.bio.ed.ac.uk/) [22]. Results were virtually independent from clock models (strict, relaxed) and tree priors (constant population size, exponential growth, Bayesian skyline). Phylogenetic affiliations of six additional isolates whose genomes had not been sequenced were determined by sequencing informative SNPs (Table S3).

## Results

### Study Population

By the end of the study period (August 2010), 32 neonates had tested positive for MRSA, *spa* type *t032* (Table S3). The attack rate was 25% (17/68) among infants of very low birthweight (VLBW; birthweight <1,500 g), in contrast to 4% (32/745) among all new admissions, yielding a relative risk of 17 (95% CI 8.1–35.5) associated with VLBW. Five neonates (16%) showed signs of an MRSA infection, including two cases of septicaemia, one pneumonia and two cases of conjunctivitis. In the case-control study, we included 23 infants who fulfilled the case definition, had a patient record and for whom we identified at least one fitting control (n = 37). One additional case had been included initially, but was excluded after genome sequencing had indicated this patient’s MRSA to be unrelated (see below). The median time between admission and the first positive MRSA swab was 8 days (range, 2 to 91 days). The median length of stay at the neonatology unit (single stay) was 47 days and did not differ significantly from controls (37 days). In September, staff screening identified two health-care workers (HCW A and B) as being colonized with MRSA, *spa* type *t032*.

### Risk Factor Analysis

Risk factors for MRSA transmission are summarized in Table 2. Most strikingly, each additional infant with the status “unknown MRSA-positive” increased the odds for other patients on the same ward to also acquire MRSA (OR = 2.5, p = 0.003). In contrast, the presence of infants with the status “known MRSA-positive” was not a risk factor (OR = 1.0, p = 0.24). Further, the number of infants cared for by each nurse on duty (ranging from 1.2 to 4.4) was associated with the risk of MRSA acquisition (p = 0.04), and moreover, contact with a specific nurse (HCW A) significantly increased the risk of MRSA acquisition (p = 0.03).

**Table 2:** Risk factor analysis in univariable logistic regression.

Variable	Odds-Ratio and 95% CI	p-value
Additional unknown MRSA-positive infant on ward	2.5 (1.26–7.99)	0.003
Contact with HCW A	9.3 (1.24-Inf)	0.03
Increase of infant-to-staff ratio by 1 unit	2.8 (1.06–9.34)	0.04
Additional unknown MRSA-positive infant in room	4.2 (0.98–197)	0.06
Peripheral venous line	0.1 (0–1.11)	0.07
Episodes of bradycardia	4.7 (0.89–47.5)	0.07
Blood transfusion	6.9 (0.72–335)	0.12
Number of X-ray treatments	0.6 (0.27–1.15)	0.16
Gastric tube	5.6 (0.62–276)	0.18
Per known MRSA-positive infant on ward	1.0 (0.97–1.13)	0.24
Number of sonographies	1.2 (0.75–1.86)	0.54
Mechanical ventilation with intubation	0.9 (0.69–1.21)	0.60
Parenteral nutrition	0.4 (0.04–3.91)	0.63
Antibiotic therapy during exposure	0.7 (0.13–3.31)	0.82
Sum of oral medications	1.1 (0.60–2.11)	0.86
Central venous line	1.4 (0.02–118)	1
Skin-to-skin (‘kangaroo’) care	0.8 (0.18–3.47)	1
Physiotherapy	1 (0.4-Inf)	1

Significant findings (5% level of confidence) and some selected variables previously reported as risk factors for MRSA transmission are shown. Ordered by statistical significance.

### MRSA Genome Diversity

**Figure 2:**
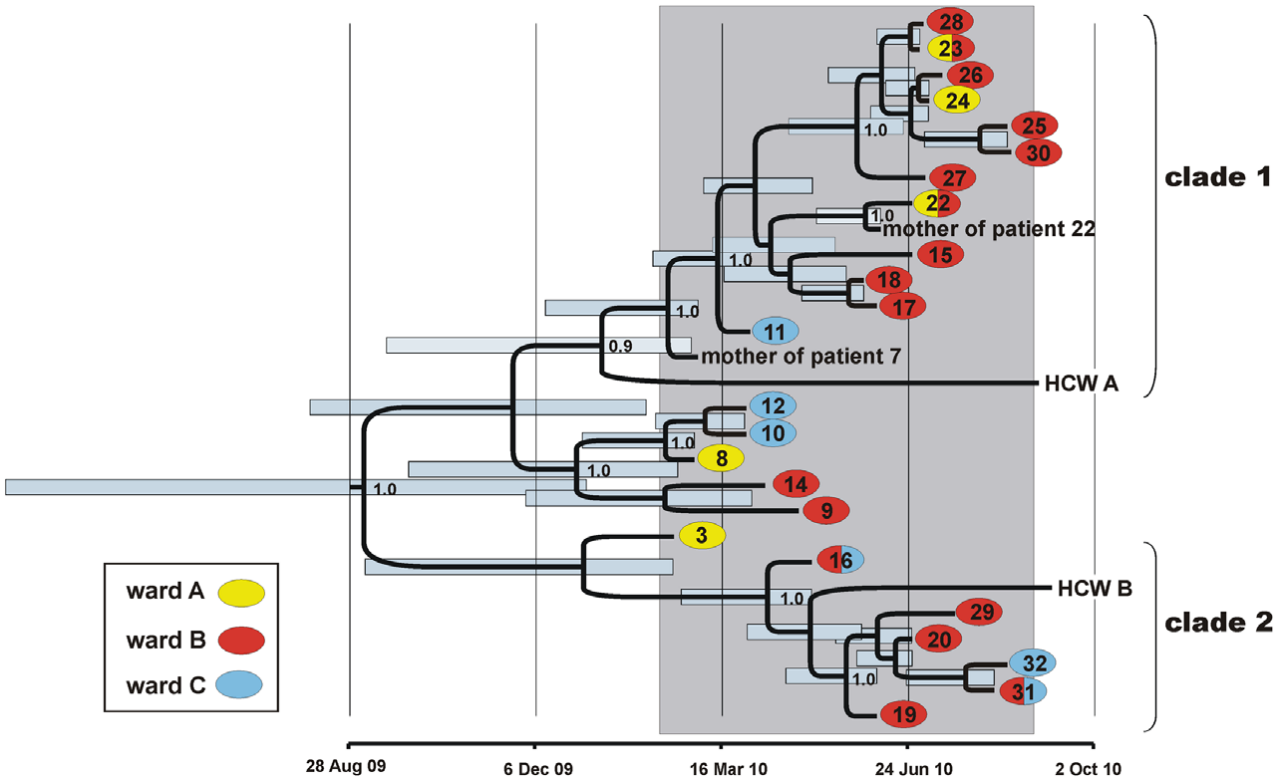
Maximum clade credibility tree based on BEAST analysis of MRSA genome sequences. Tips of the tree are constrained by bacterial isolation dates, the time scale is shown at the bottom. Node support is indicated for posterior probabilities ≥0.9. The case-control study period (February 8 to August 31, 2010) is indicated by grey shading. MRSA from patients (patient numbers are indicated), healthcare workers (HCW A, HCW B) and two mothers of patients are included. Colours indicate patient positions on wards A, B, and C, respectively. Blue bars indicate 95% Bayesian credibility intervals of bacterial divergence dates (node heights).

We determined genome sequences from 30 MRSA isolates collected during the study period. These included 24 isolates from patients initially included in the case-control study, four isolates from two additional patients and their nasally colonized mothers, respectively, and two isolates from colonized health-care workers (HCW A and B; Table S3). Phylogenetic analysis on the basis of detected SNPs indicated that 28 isolates formed a strongly supported monophyletic clade (Figure 2, Figure S1), confirming close epidemiological linkage between outbreak isolates. In contrast, two isolates (10-02187, 10-02193) fell outside this clade and were equally divergent from the predominant strain as from each other, as well as from another ST22 isolate from the UK (HO50960412 [21]) (>100 SNPs in binary comparisons; Figure S1). We conclude that in addition to the predominant strain, two independent ST22 strains were present in the neonatology unit during the case-control study period, which had not been recognized previously on the basis of conventional (*spa*) typing. The patient with isolate 10-02193 had been included in the case-control study initially, but was removed from the dataset once the MRSA genome sequence had indicated it was unrelated.

In the core genomes from 28 outbreak isolates, we identified a total of 26 SNPs (Table S2), which were verified by PCR and chain-termination sequencing. This limited DNA sequence diversity resulted in a remarkably complex phylogenetic tree (Figure S1). The root-to-tip distances of individual isolates in this tree correlated positively with the dates of isolation (p<0.001; Figure S2), indicating that the bulk of the observed DNA sequence variation had accumulated during the study period, i.e. within only seven months.

### Testing Hypotheses on Transmission Pathways

We exploited MRSA genome variation to test several hypotheses about potential MRSA transmission events, which had been provided through our epidemiological investigation:

1 Because of the close contact between neonates and their mothers, transmission between them was presumed in two cases, where both had tested positive for MRSA *t032* (Table S3).
In the first case, MRSA genomes recovered from infant and mother were fully identical, confirming the epidemiological linkage (isolates 10-02172, 10-02737; Figure S1). In the second case, however, the MRSA from the infant (10-02187) was unrelated to the predominant strain and clearly disparate from her mother’s isolate (10-02739), indicating that a transmission between mother and infant had not occurred (Figure S1).
2 Among 160 staff members tested, contact with healthcare worker HCW A was identified as a risk factor for MRSA acquisition (Table 2). Consequently, colonisation of this person with MRSA *spa* type *t032* suggested her nasal staphylococcal flora as a source for repeated transmission to multiple patients.
Isolate 10-02735, which was recovered from HCW A on 2nd September 2010, descends from a basal position within clade 1 of the phylogenetic tree (Figure S1), and according to our coalescence-based analysis the most recent common ancestor of 10-02735 and other isolates in clade 1 dates back to 9th January 2010 (95% confidence interval, 17th September 2009 to 27th February 2010; Figure 2). Since then, 10-02735 has accumulated four point mutations that were not found in any patient isolates (Table S2), and, in turn, patient isolates in clade 1 carry one to eleven additional mutations that were not found in 10-02735. Hence, the MRSA colonising this healthcare worker has evolved independently from the patients’ colonising strains since approximately January 2010, and MRSA genomes provide no evidence of reciprocal exchange between patients and staff since then.
3 By integrating epidemiological (temporal, spatial) and genetic data, it was possible to reconstruct probable transmission chains that are represented by MRSA isolates in phylogenetic clade 1 and clade 2, respectively (Figure 2).
Based on the relative timing of MRSA detection in the different patients and their spatial proximity alone, patient 19 could have acquired the MRSA (isolate 10-02176) in June 2010 from patients 9 or 14 (who at the time were in a different room on ward B; Figure 2, Figure S3) or from patients 18 or 13 (on ward A; Figure 2, Figure S3). However, MRSA isolates from these patients are affiliated with different clades in the phylogenetic tree (Figure 2, Table S1) and, consequently, the most recent common ancestor shared with 10-02176 from patient 19 dates back almost one year (95% confidence interval, 23rd February 2009 to 3rd January 2010; Figure 2). Hence, they can be ruled out as sources for direct transmission to patient 19 based on phylogeny, which rather suggests 10-02179 (patient 16) and 10-02736 (HCW B) as ancestors of 10-02176 (Figure 2). Twenty-eight days had passed between positive MRSA tests for patients 16 and 19 (Figure S3), making direct transmission between the two patients unlikely. However, both epidemiology and phylogeny are consistent with a scenario, where nasally colonized healthcare worker HCW B, from which isolate 10-02736 was recovered in September (Table S3), may have contracted the MRSA from patient 16 and served as a vector for transmission to patient 19. The phylogenetic position and divergence time of isolate 10-02736 indicates that HCW B had been colonized with MRSA already in May (95% confidence interval, 31st March to 28th May 2010; Figure 2), when otherwise there were no closely related MRSA on the ward.

On the basis of temporal and spatial data, it is likely that the MRSA was subsequently transmitted from patient 19 to patient 20 (who was in the same room when turning MRSA-positive) and to patient 29 (in a different room), from where it was then passed on to patient 31 (who shared a room with patient 29). While MRSA-colonized, patient 31 was transferred to Ward C (Figure S3), and his MRSA apparently got transmitted to patient 32 (who was in a different room on Ward C). This scenario is consistent with genome sequences from the respective isolates, which are fully identical except for three mutations in the genome from isolate 10-02165, whose neonate host (patient 29) had developed bacteremia (Figure 2; Table S2).

Clade 1 in the phylogenetic tree (Figure 2) seems to represent another transmission chain, even though the precise series of events is less clear in this case. Isolates 10-02177 and 10-02178, whose genomes are identical, are from patients 17 and 18 in the same room, suggesting transmission. Additional patients successively acquired MRSA when they were on Ward B together with colonized patients 17 and 18 (Figure S3), yielding closely related MRSA isolates (10-02169, 10-02161, 10-02163, 10-02166, 10-02168, 10-02170, 10-02171) (Figure 2, Figure S1), again suggesting repeated transmission events.

## Discussion

### Risk Factors for MRSA Transmission

Our study identified a number of risk factors for MRSA transmission, of which very low infant birth weight [1], [6], [23] and understaffing [24] had been reported previously. In addition, however, we detected an increased risk of transmission from “unknown MRSA-positive” infants on ward, but not from “known MRSA-positive” infants (Table 2). The strong association of “unknown MRSA-positive” patients with transmission of MRSA was further underscored by a dose-response relationship, hinting at a causal effect. To our knowledge, the definition of the status “unknown MRSA-positive” is a new concept. It is based on the delay from taking a swab to receiving the result on the ward - usually two days in our case. During this period, an infant who turned out later to be MRSA-positive posed the highest risk to others. This is a plausible finding because known MRSA-positive infants were cared for as a separate cohort, while new patients with unknown MRSA status were treated together with MRSA-negative infants. This result suggests that shortening the time span between swabbing and receiving bacteriological results may help to reduce cross infections with MRSA in settings where isolation of all new admissions is not possible. It also suggests that staff complied with hygiene standards better when dealing with a known MRSA-positive infant.

Healthcare workers colonized with MRSA may constitute a source for nosocomial infections [25], [26]. In our study, two healthcare workers tested positive for MRSA *t032*, and one of them (HCW A) was associated with an increased risk for MRSA transmission in the case-control study (the only one out of 160 staff members tested). However, the MRSA isolate recovered from HCW A displayed a number of genomic differences to those from patients, making the nasal flora of this person an unlikely reservoir for MRSA transmission during the study period (Figure 2). Hence, the increased risk associated with HCW suggests a role in transmitting MRSA from infant to infant. It is conceivable that the same practices that facilitate transmission between patients could also promote self colonization. However, it cannot be ruled out completely that the statistical significance found is due to chance alone. In contrast, through transfer of MRSA to a single patient, nasal colonization in HCW B may have sustained transmission during a period without MRSA-positive patients on ward.

### MRSA Genomes Document Transmission History

Two recent studies demonstrated that genome sequences from sets of MRSA isolates could be generated and analysed within few days by using the latest generation of benchtop sequencing machines, enabling unequivocal identification of strains causing outbreaks and of other strains that were unrelated [20], [21]. In our study, the latter result was exemplified by two isolates (10-02193, 10-02187) that were identified as being unrelated to the predominant strain, even though they had been indistinguishable by conventional typing (Figure S1).

Moreover, our results demonstrate that microevolution of MRSA proceeded fast enough to mirror MRSA transmission history within a single hospital unit, over the course of few months. MRSA genomes had accumulated sufficient variation to test epidemiological linkage among individual patients, between infants and their mothers, and between patients and staff members. Genome sequences documented likely transmission events between patients that had shared rooms, but also between different rooms on the same ward, supporting the case-control study result which indicated that the risk of MRSA transmission increased with each unknown MRSA-positive infant on the ward (Table 2). Further, MRSA spread between wards associated with patient transfer was detected and the relevance of individual nurses’ nasal colonization could be evaluated.

One limitation of our study is that the diversity of MRSA within individual hosts was not measured, as multiple isolates from single patients or staff had not been collected. Little is known about intra-host variation of MRSA genomes, which could potentially result in uncertainties of transmission reconstructions. Such uncertainty will likely be greatest when the genetic distance between isolates is particularly small, exemplied in our study by ≤2 SNPs among MRSA from infants sharing rooms.

In our sample of extremely closely related MRSA genomes, point mutations had accumulated at 2.4×10^−6^ nucleotide substitutions per nucleotide site and year on average (95% confidence intervals, 1.3×10^−6^ to 3.6×10^−6^), which is very similar to the short-term evolutionary rates previously found for other MRSA populations that had been collected over much wider time spans and geographical ranges [18], [19]. This rate corresponds to approximately one mutation per genome every six weeks. Accordingly, and due to the stochastic occurrence of mutations, we found several genomes that were indistinguishable even though they had been sampled from different patients up to 70 days apart (isolates 10-02162, 10-02176). In contrast to a recent report [21], we did not observe any hyper-mutators. However, both isolates sampled from bacteremia (10-02165, 10-02169) sat at conspicuously long branches in the phylogenetic tree (Figure S1), each caused by two or three unique mutations, respectively. This preliminary result suggests that MRSA evolution may accelerate during bloodstream infection, which was proposed only recently [27], and warrants systematic investigation.

Taken together, MRSA genome sequencing proved a powerful tool for testing several hypotheses on specific MRSA transmission routes within the neonatology unit. In the light of recent advancements of sequencing technologies and rapidly declining sequencing costs, our result opens exciting prospects for genome-based epidemiological investigations of MRSA spread at a local level, where conventional typing techniques commonly lack discriminatory power due to the predominance of very few genotypes [28], [29]. Genome sequencing has proven useful for investigating the epidemiology of other bacterial pathogens, too [30], and has the potential to become a routine tool in clinical bacteriology [31]–[33].

## Supporting Information

Figure S1 **Maximum-likelihood phylogenetic tree.**
(PDF)

Figure S2 **Correlation of root-to-tip distances from the maximum-likelihood phylogenetic tree vs. isolation dates.**
(PDF)

Figure S3 **Chart indicating room numbers for patients during the case-control study period and their retrospective MRSA status.** Colour-filled squares indicate days when infants were MRSA-positive, and colours indicate wards (yellow, red, and blue for wards A, B, and C, respectively). Empty squares without numbers indicate days when infants were not registered on any of the three wards. The table on the left indicates which patients were included in the case-control study. (This figure is meant to be looked at on screen, where it can be zoomed).
(XLS)

Table S1 Bacterial isolates.
(XLSX)

Table S2 Single-nucleotide polymorphisms.
(XLSX)

Table S3 PCR primers.
(XLSX)
