# Supplementary figures and images for: MRSA Transmission on a Neonatal Intensive Care Unit: Epidemiological and Genome-Based Phylogenetic Analyses
Source: PLoS One. 2013 Jan 31;8(1):e54898. doi: 10.1371/journal.pone.0054898 (PMC3561456; doi:10.1371/journal.pone.0054898)

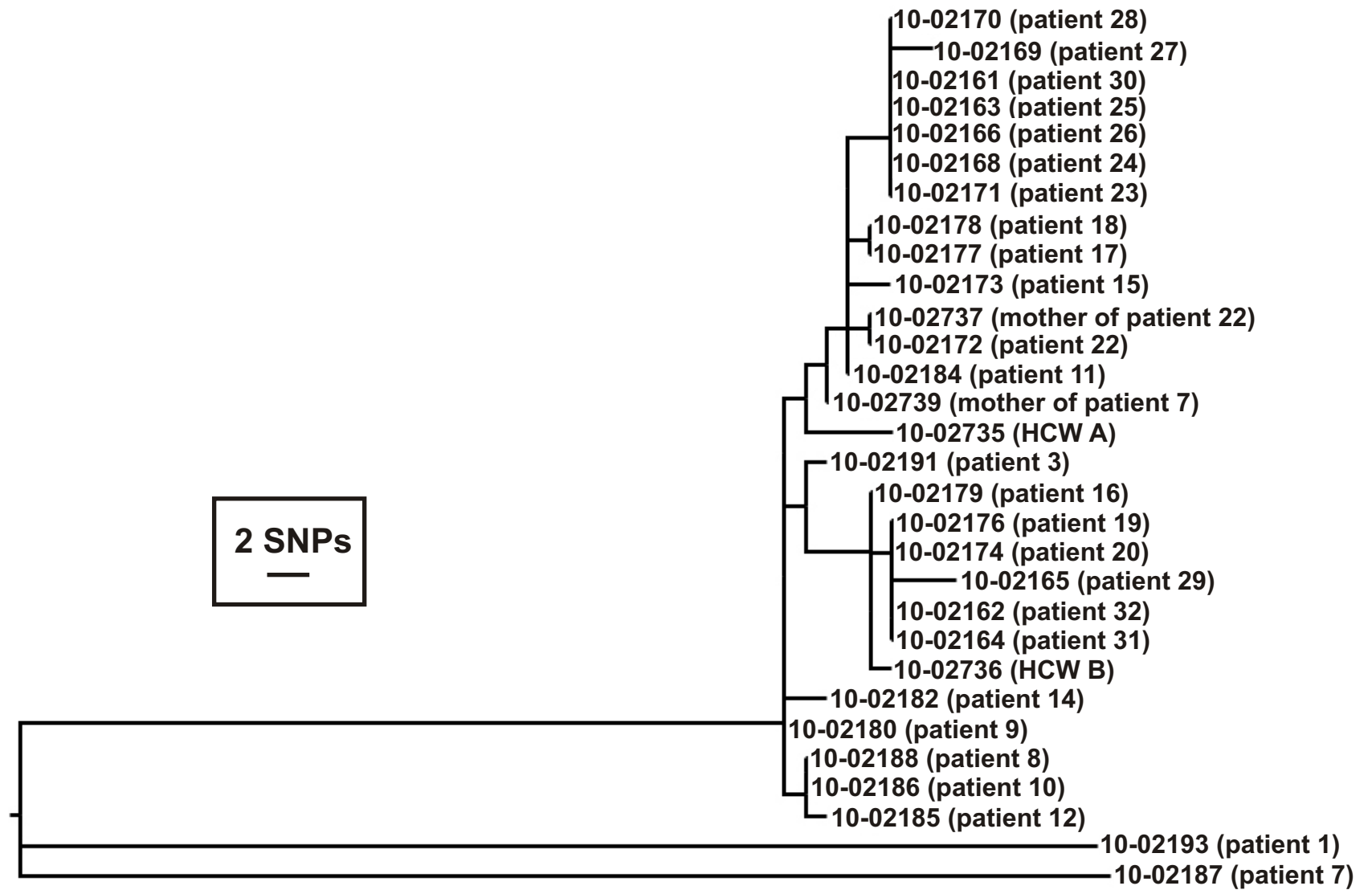

Supplement: Figure S1 — Maximum-likelihood phylogenetic tree. (PDF) [file pone.0054898.s001.pdf]

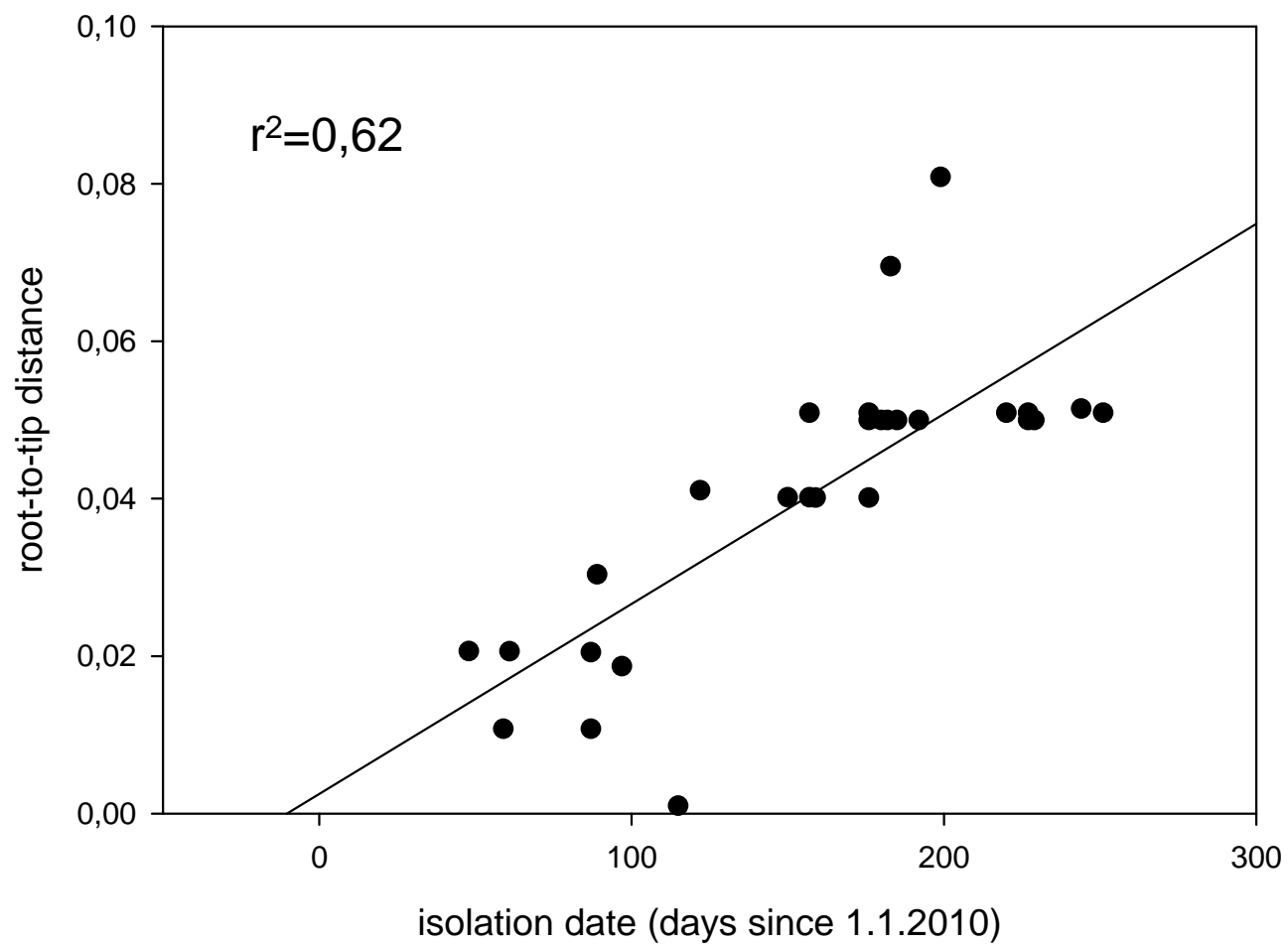

Supplement: Figure S2 — Correlation of root-to-tip distances from the maximum-likelihood phylogenetic tree vs. isolation dates. (PDF) [file pone.0054898.s002.pdf]
